# Modernizing Reference Genome Assemblies

**DOI:** 10.1371/journal.pbio.1001091

**Published:** 2011-07-05

**Authors:** Deanna M. Church, Valerie A. Schneider, Tina Graves, Katherine Auger, Fiona Cunningham, Nathan Bouk, Hsiu-Chuan Chen, Richa Agarwala, William M. McLaren, Graham R.S. Ritchie, Derek Albracht, Milinn Kremitzki, Susan Rock, Holland Kotkiewicz, Colin Kremitzki, Aye Wollam, Lee Trani, Lucinda Fulton, Robert Fulton, Lucy Matthews, Siobhan Whitehead, Will Chow, James Torrance, Matthew Dunn, Glenn Harden, Glen Threadgold, Jonathan Wood, Joanna Collins, Paul Heath, Guy Griffiths, Sarah Pelan, Darren Grafham, Evan E. Eichler, George Weinstock, Elaine R. Mardis, Richard K. Wilson, Kerstin Howe, Paul Flicek, Tim Hubbard

**Affiliations:** 1 National Center for Biotechnology Information, National Library of Medicine, National Institutes of Health, Bethesda, Maryland, United States of America; 2 The Genome Institute at Washington University, St. Louis, Missouri, United States of America; 3 The Wellcome Trust Sanger Institute, Wellcome Trust Genome Campus, Hinxton, Cambridge, United Kingdom; 4 The European Bioinformatics Institute, Wellcome Trust Genome Campus, Hinxton, Cambridge, United Kingdom; 5 Department of Genome Sciences, University of Washington School of Medicine, Seattle, Washington, United States of America; 6 Howard Hughes Medical Institute, Seattle, Washington, United States of America

## The Rationale for the GRC

The availability of a high quality human genome assembly has revolutionized biomedical research. Genomics has now entered the realm of clinical genetics, with many groups using either whole genome sequencing [1],[2] or whole exome sequencing [3] to identify variants underlying diseases and informing treatment options [4]. Advances in technology have increased the number of sequenced human genomes; however, de novo assembly of next generation sequencing reads is still problematic. The alignment of sequencing reads from these new genomes to a high quality reference genome remains a critical aspect of data interpretation [5].

While the human reference assembly is the highest quality mammalian assembly available, it is not without shortcomings. The “finished” assembly [6] contained over 300 gaps in the euchromatic portion of the genome, tiling path errors and regions represented by uncommon alleles. Furthermore, assessment of genome-wide variation revealed regions of the genome with complex, structurally diverse, allelic representations [7]–[9] that were insufficiently represented in the reference genome. Other analyses identified sequences that failed to align to the reference assembly either because the reference assembly contained a valid deletion allele or underrepresented multi-copy genes [10]–[13]. The Genome Reference Consortium (GRC) was formed to address these issues.

The GRC (the GRC consists of The Genome Institute at Washington University, The Wellcome Trust Sanger Institute, The European Bioinformatics Institute, and The National Center for Biotechnology Information) is an international consortium with expertise in genome mapping, sequencing, and informatics. The goal of the GRC is to provide high quality genome assemblies that will allow a user to place any sequence greater than 500 bp into a chromosome context. While this report focuses largely on recent GRC advances concerning the human reference assembly, the GRC is also responsible for the mouse and zebrafish reference assemblies. Continued improvement of the human reference assembly is critical as we move towards an era of clinical and personal genomics. The reference genomes of mouse and zebrafish are similarly critical in light of their importance as model organisms and the significant investments made in creating community resources such as gene knockout collections.

## Assembly Management

Two major problems faced the GRC at the outset of this project, the decentralized nature of the Human Genome Project and the lack of a suitable data model for representing complex genomes. Much of the data underlying curation decisions had not been captured nor standardized. The human reference assembly had never been submitted to the International Nucleotide Sequence Database Collaboration (INSDC) [14] and thus lacked stable, trackable sequence identifiers that could be accessed from any INSDC database.

Initial efforts at assembling the human genome were guided by the concept of “a golden path” [15], a single clone tiling path that could be reduced to one non-redundant haploid representation of the human genome. While this model fit well with the prediction that single nucleotide variants (SNVs) would be the predominant source of variation in the population, it is now clear that structural variation is a much larger source of genomic diversity than previously recognized [16],[17]. Additionally, this model did not deal robustly with sequences that were not part of chromosome assemblies. These often represent sequences that cannot be easily ordered or oriented on the chromosome assembly due to structural complexity but frequently contain genes that may be of biological interest [18] or represent alternate haplotypes of regions in the chromosome assembly [9],[19]. Earlier versions of the reference genome assembly included some of these allelic variants (such as at the MHC region) but the sequences themselves often were not used because they had no relation to the chromosome sequence and could not be easily distinguished from sequences reflecting biological or artificial duplication.

The GRC has addressed these problems by establishing common tools and standard operating procedures (SOPs) so that the genome assembly is now constructed in a regularized fashion. We have developed a single database to store all data underlying the genome assembly. Finally, we have developed a system to track individual regions that are under review. All of these data are made publicly available through our Web site (http://genomereference.org/).

**Figure 1:**
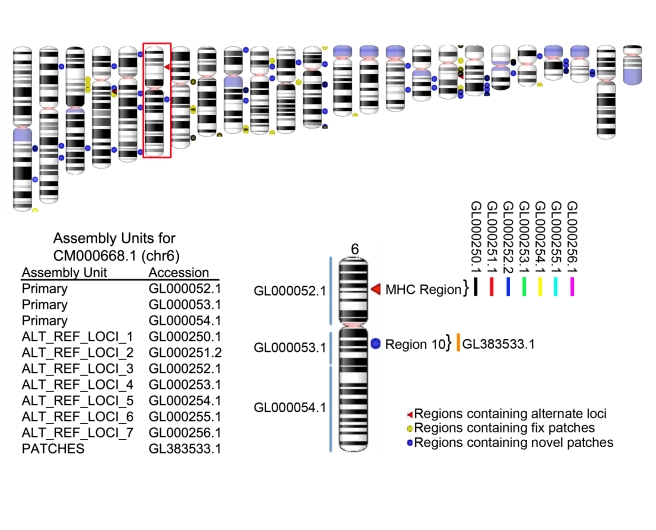
Assembly representation for GRCh37.p3. The top panel shows an ideogram representation of the human genome. The primary assembly unit contains sequences for the non-redundant haploid assembly; this includes the scaffolds that make up the chromosome sequence as well as unplaced and unlocalized scaffolds that are thought to represent novel sequence (not shown in this picture). Alternate loci and patches are placed in separate assembly units to facilitate annotation. Note the seven alternate scaffolds in the MHC region are all placed in different assembly units, as they all represent different representations of the same sequences. Other alternate loci can be added to these assembly units at the next major release if they don’t overlap the existing alternates. All patches are placed in the PATCHES assembly unit and minor releases are cumulative such that the latest minor release will contain all patches. The red triangle, yellow circles, and blue circles represent regions that contain additional sequences that are not given actual chromosome coordinates, but rather are given a chromosome context via alignment to the primary assembly. The red triangles represent regions’ alternate loci; these are sequences that provide an additional tiling path to the one given in the chromosome representation and are essential for representing structurally complex loci. The circles represent patch sequences; these are minor updates made to the assembly outside of the major build cycle. Yellow circles represent “fix” patches: regions of the chromosome assembly that will change with the next major assembly update. Blue circles represent “novel” patches: these are sequences that represent new alternate loci in the next major assembly update. Unlocalized and unplaced sequences are not represented in this figure. Sequences within the assembly are placed within containers known as assembly units. Note: a region can point to more than one type of extra chromosomal sequence; for example, a region could point to an alternate locus and to a fix or novel patch.

Additionally, the GRC has formalized an assembly model (Figure 1 and Box 1) that provides for improved accounting for all sequences, including those that are not part of chromosome assemblies, and facilitates genome annotation by placing additional structure on those sequences. Structurally complex regions can be represented by more than one tiling path; one of which will be integrated into the chromosome assembly while the others will be instantiated as an independent sequence that, by alignment to the chromosome, provides the chromosome context for the alternate allele.

**Box 1.** Assembly Definitions

**AGP:** A file used to describe the instructions for building a contig, scaffold, or chromosome sequence. This file specifies the order, orientation, and switch points for each component.

**Alternate Locus:** A sequence that provides an alternate representation of a locus found in a largely haploid assembly. These sequences don’t represent a complete chromosome sequence, although there is no hard limit on the size of the alternate locus; currently these are less than 5 Mb.

**Assembly:** A set of sequences (chromosomes, unlocalized, unplaced, and alternate loci) used to represent an organism’s genome.

**Assembly Unit:** Collections of sequences used to define discrete parts of an assembly.

**Component:** The basic genomic level sequence used to construct the genome; typically these are clone sequences, Whole Genome Shotgun sequences, or PCR fragments. These sequences must be submitted to GenBank/EMBL/DDBJ.

**Contig:** A contiguous sequence generated from determining the non-redundant path along an ordered set of component sequences. A contig should contain no gaps.

**Patch:** A genome patch is a scaffold sequence that is part of a minor genome release. These sequences either correct errors in the assembly (a FIX patch) or add additional alternate loci (a NOVEL patch). These sequences allow us to update the assembly information without disrupting the chromosome coordinate system. FIX patches will be removed at the next major assembly release, as the changes will be rolled into the new assembly. NOVEL patches will be moved from the PATCHES assembly unit to a proper assembly unit.

**Primary Assembly Unit:** Represents the collection of sequences that, when combined, represent a non-redundant haploid genome.

**Scaffold:** An ordered and oriented set of contigs. A scaffold will contain gaps, but there is typically some evidence to support the contig order, orientation, and gap size estimates.

**TPF:** Tiling Path File; this provides the order of the component sequences that are used to build a higher order sequence (contig, scaffold, or chromosome).

**Switch Point:** The base at which the contig sequence stops being generated from one component sequence and switches to using the next component sequence. There must be at least one switch point between adjacent component sequences in a contig.

**Unlocalized sequence:** A sequence found in an assembly that is associated with a specific chromosome, but that cannot be ordered or oriented on that chromosome.

**Unplaced sequence:** A sequence found in an assembly that is not associated with any chromosome.

We have also introduced the concept of a “minor” assembly update, in the form of genome patches. This mechanism provides users with timely access to genome improvements without inducing frequent changes to the coordinate system upon which assembly annotations are based. Because genome patches take the same form as alternate loci the two forms of data can be similarly managed.

The release cycle for major assembly updates will not occur on a fixed schedule. In order to minimize the need for frequent re-annotation, major assembly updates will occur infrequently when we have produced at least 100 fix patches or affected >1% of the euchromatic sequence. The GRC will announce planned updates on their Web site at least 6 months in advance of any major assembly release. Additional, detailed information regarding major releases will be publicly announced via the Web site as data freeze dates approach. Minor assembly updates will be made quarterly.

## Assembly Quality and Improvement

We have produced a major release of the human reference assembly, GRCh37, which was submitted in June of 2009 to the INSDC (GCA_000001405.1), and four minor assembly updates, with the last patch, GRCh37.p4 (GCA_000002405.5), released in April 2011. Detailed information concerning genome assembly construction is on our Web site (http://www.ncbi.nlm.nih.gov/projects/genome/assembly/grc/info/index.shtml).

**Figure 2:**
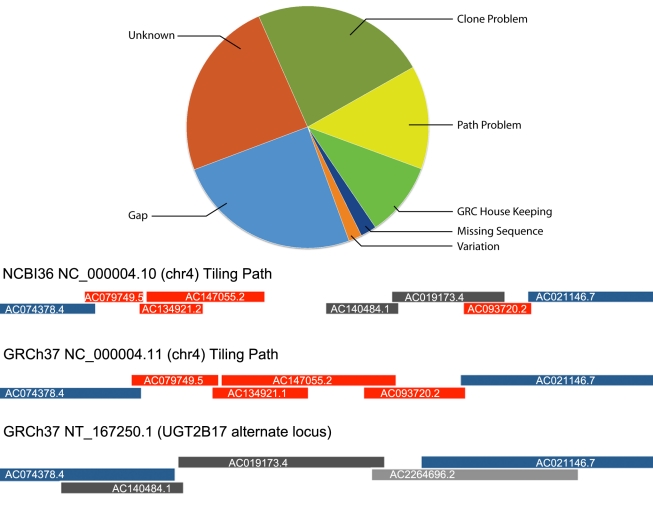
Distribution of issues addressed and an example region. (Top Panel) Issues for GRCh37, GRCh37.p1, and GRCh37.p2, broken down by type. Issue types are: Clone Problem: The issue is contained within a single clone. This may be a single nucleotide difference or a clone mis-assembly. Path Problem: There is evidence that the tiling path within a given region is incorrect and we will need to update the path. GRC Housekeeping: Changes use to help regularize the tiling path. Missing Sequence: Sequence that we can’t yet place on the assembly. Mapping studies are ongoing to help place these sequences. Variation: There is evidence to suggest that complex variation is complicating a region and an alternate allele may need to be produced. Gap: The issue concerns filling a gap. Unknown: Issue is still under investigation for classification. (Bottom Panel) Details for issue HG-2, a Path Problem. The representation in NCBI36 was a mixed haplotype. The tiling paths for NCBI36 and GRCh37 are shown. Blue clones are anchor clones that are in NCBI36, the GRCh37 chr4 path, and the GRCh37 alternate locus path. Red clones represent the UGT2B17 insertion path and dark gray clones represent the UGT2B17 deletion path. The light gray clone was not used in NCBI36, but was used in GRCh37 to complete the alternate locus.

The top part of Figure 2 shows the distribution of issue types that were resolved for these assembly releases. Some assembly updates are relatively minor, involving the correction of a single nucleotide discrepancy in the assembly (e.g., HG-445; http://www.ncbi.nlm.nih.gov/projects/genome/assembly/grc/issue_detail.cgi?id=HG-445) while others involved multiple components and required generation of new, region-specific tiling paths (e.g., HG-2; http://www.ncbi.nlm.nih.gov/projects/genome/assembly/grc/issue_detail.cgi?id=HG-2). (Figure 2) [20].

While the model changes described above facilitated our assembly management and reporting, we also wished to investigate whether these updates would allow for improved genome analysis. To investigate this, we first tried to recover sequence identified as novel in a personal genome, theYH1 human assembly [12]. Roughly 25% could be placed in a chromosome context using GRCh37.p2 (see supplemental table 1 and supplemental figure 1 at http://www.ncbi.nlm.nih.gov/projects/genome/assembly/grc/supplement/). The remaining sequences are being investigated to determine if they warrant inclusion in a future assembly release.

We also wished to investigate the impact on alignment of next generation sequencing reads. We selected two samples from the 1,000 Genomes project [21], NA12156 and NA12878, (SRA accessions ERX000125 and ERX000080, respectively) and aligned their reads to GRCh37, with and without the alternate loci. We demonstrated that removal of the alternate loci leads to misalignment of approximately two-thirds of the alternate-locus specific reads (see supplemental table 2, supplemental figure 2 at http://www.ncbi.nlm.nih.gov/projects/genome/assembly/grc/supplement/). These data clearly demonstrate that that inclusion of alternate representations for genomic loci can improve alignment quality and thus avoid spurious variation calls.

## Policy Implications

We envision the high quality reference assemblies generated by the GRC having a long-term role in biomedical research because they most accurately capture all forms of human genetic variation and facilitate investigation of human disease in model organisms. With this in mind, we have built a reference assembly infrastructure to support transparent curation and assembly production. We have also updated the assembly model so that it better represents our current understanding of genome structure and diversity. We will use this model to encompass new discoveries and ultimately capture all significant variations in the human population structure as discovered through projects such as 1,000 genomes. Additionally, we wish to engage the research and clinical communities to identify regions that require targeted effort and to incorporate information from groups performing detailed work on specific loci. The GRC can only be truly successful with community input. Users can report problems directly to the GRC via our Web page (http://www.ncbi.nlm.nih.gov/projects/genome/assembly/grc/ReportAnIssue.shtml).

It is difficult to overstate the importance of the human reference assembly, even in the age of personal genomics. Given current sequencing and assembly technology, there is a clear need for a high quality reference that can represent structural diversity across all populations. Providing a representation of this diversity is critical for next generation sequence analysis. Even using an assembly with only three regions with alternative alleles, we show improved alignment quality and by extension variation calling, which is the primary product of personal genomics. More genomic alignment tools that can take the alternate representations into account need to be developed.

Understanding how genotype influences phenotype necessitates an accurate and complete picture of all loci in multiple populations. For many genomic regions, this can be denoted by a sequence with annotated SNPs and small indels, but other loci will require multiple sequence instances for complete representation. Some human loci, such as the 1q21 region, which remains misassembled in GRCh37.p2, are sufficiently complex that significant effort is needed to obtain even one correct sequence for the region. Additional work is required to sort out the haplotypes segregating among various populations, many of which contribute to phenotypes associated with multiple developmental disorders [22].

While assemblies using next generation sequencing are beginning to approach the quality of long-read Whole Genome Shotgun assemblies [23], they continue to fail in complex regions. While it is likely that sequencing and assembly technology will improve such that de novo assembly of individual genomes will approach the quality of the human reference, it is not clear when this will happen. However, even when this is a common occurrence, we see a role for the GRC in integrating the data from thousands of human genomes to produce a “gold-standard” reference assembly. We anticipate a continued need for a high quality reference assembly that will allow any human sequence to be placed into a chromosome context quickly and easily. As we march down the path of personal genomics it is critical that we devote resources to the current reference assembly in order to support clinical applications. As we continue to understand how genotype influences phenotype, the best possible reference assembly available must be made available to the research community.

## Abbreviations

GRC: Genome Reference Consortium
INSDC: International Nucleotide Sequence Database Collaboration
